# The hub-and-spoke organization design revisited: a lifeline for rural hospitals

**DOI:** 10.1186/s12913-017-2755-5

**Published:** 2017-12-13

**Authors:** James K. Elrod, John L. Fortenberry

**Affiliations:** 1 Willis-Knighton Health System, 2600 Greenwood Road, Shreveport, LA 71103 USA; 2 LSU Shreveport, 1 University Place, Shreveport, LA 71115 USA

**Keywords:** Hub-and-spoke, Organization design, Rural hospitals, Medically underserved populations

## Abstract

**Background:**

Characterized by declining populations, high poverty, reduced employment opportunities, and high numbers of uninsured residents, rural communities pose significant challenges for healthcare providers desirous of addressing these medically underserved areas. Such difficult environments, in fact, have forced the closure of many rural hospitals across America, with scores facing the same threat, compelling intensive efforts to identify pathways which will yield an improved future.

**Discussion:**

Collaborations with stronger urban or suburban healthcare institutions offer a prudent avenue for rural hospitals to continue serving their patients. Such relationships can be structured in many different ways, but Willis-Knighton Health System found that its use of the hub-and-spoke organization design set the stage for the institution to cast a vital lifeline to neighboring rural hospitals, affording the relatively seamless integration and assimilation of partner facilities into its network, ensuring continuity of services in remote regions. This article supplies an overview of the hub-and-spoke network and discusses Willis-Knighton Health System’s use of it to facilitate the establishment of productive partnerships with rural hospitals.

**Conclusions:**

The delivery of healthcare services in rural environments is essential, but with small community hospitals increasingly being under threat, the outlook is not particularly attractive. Partnerships with better positioned healthcare entities offer significant hope, but care must be taken to structure these arrangements optimally. Willis-Knighton Health System found utility and value in its hub-and-spoke organization design, with the insights presented in this account potentially offering a pathway for others to follow as they go about addressing the healthcare needs of rural populations.

## Background

Rural populations are among the most vulnerable in America [1–4]. They are poorer, older, and sicker than their counterparts residing in densely-populated areas, and the communities where they live are increasingly losing an already compromised pool of healthcare resources [5, 6]. Convenient access to healthcare services in these small communities was commonplace at one time, but the increasing urbanization and suburbanization of society has taken a severe toll on the viability of rural America, reducing population, the tax base associated with such, and related public and private investment. High poverty, reduced employment opportunities, and high numbers of uninsured residents further characterize and burden rural communities [7]. These consequences understandably have negatively impacted community infrastructure, notably including the availability of healthcare services, their depth and breadth, and their accessibility to area residents. In recent decades, rural hospitals, the traditional backbone of healthcare delivery in small communities, have been closing at a very concerning rate [6]. In fact, according to the Cecil G. Sheps Center for Health Services Research, 81 rural hospitals in America have been shuttered since January 2010 [8]. Further, there are strong indications that this crisis will escalate, with an estimated 673 rural hospitals being considered to be vulnerable for closure [6, 9, 10]. This places hardships not only on providers of care and their associated employees, but also and most importantly on those residing in rural communities who face the daunting prospect of diminished or nonexistent healthcare access in these remote areas.

Few states have escaped the consequences of dwindling healthcare resources in their rural communities and Louisiana is no exception, with 58% of its rural hospitals facing the threat of closure [10]. Shreveport, Louisiana-based Willis-Knighton Health System, which holds market leadership in the northwest region of the state and delivers comprehensive healthcare services through multiple hospitals, numerous general and specialty medical clinics, and a range of complementary establishments, operates primarily in the region’s urban and suburban population centers. Those residing in remote areas typically look to local, independent rural hospitals for basic healthcare needs and often travel to Willis-Knighton Health System’s facilities for more intensive services. Although small and limited, the region’s rural hospitals play a vital role in the continuum of care, especially regarding their delivery of primary care, emergency services, and similar offerings at the local level. Closures would harm the populace and place increasing burdens on healthcare facilities in adjacent markets to accommodate those left without local providers.

To those who actively have been monitoring the environment, the crisis currently being experienced in rural healthcare did not occur overnight. Instead, it has been decades in the making. Willis-Knighton Health System recognized the diminishing prospects of its neighboring rural hospitals years ago and decided to take proactive steps by forming partnerships with select facilities to ensure that their medically underserved populations retained access to quality healthcare services locally. Fortuitously, Willis-Knighton Health System’s adoption of the hub-and-spoke organization design, a decision made in the early 1980s and credited for much of its success [11, 12], also set the stage for the system to cast a vital lifeline to neighboring rural hospitals, affording the relatively seamless integration and assimilation of partner facilities into the Willis-Knighton Health System network, ensuring continuity of services in remote regions. This article supplies an overview of the hub-and-spoke network and discusses Willis-Knighton Health System’s use of it to establish productive partnerships with rural hospitals, potentially offering a pathway for other health systems to follow as they seek to ensure that rural populations continue to have local healthcare options.

## Overview and attributes

The hub-and-spoke model, as applied in healthcare settings, is a method of organization involving the establishment of a main campus or hub, which receives the heaviest resource investments and supplies the most intensive medical services, complemented by satellite campuses or spokes, which offer more limited service arrays at sites distributed across the served market. Basic healthcare needs are addressed locally through the network’s satellite facilities, but in cases where more intensive medical interventions are required, patients are routed to the main campus or hub for treatment [13, 14]. The hub-and-spoke model is a highly scalable, efficient design, with satellites being added as needed or desired [14–17]. In cases where spoke-to-hub access proves impractical, additional hubs can be developed, creating a multi-hub network [15, 18, 19]. In an earlier article profiling Willis-Knighton Health System’s experiences developing its hub-and-spoke network [12], benefits associated with the model were noted as follows.

1. Consistency across operations, courtesy of policies being issued and enforced system wide by the main campus hub, offering uniformity throughout the network;
2. Increased efficiencies, as the most advanced medical technologies and expertise are centralized at the main campus, avoiding the costly duplication of services across sites, increasing return on investment, bolstering economies of scale, and reducing the costs of healthcare for patients and insurers;
3. Enhanced quality, as resources and expertise concentrated at single sites bolster patient volume, fostering improved outcomes;
4. Enhanced market coverage, as satellites, courtesy of their more limited service arrays, carry fewer resource requirements, facilitating expansion initiatives when and where needed, affording an extremely high degree of scalability; and
5. Improved agility, as the synergies associated with operational consistency, efficiencies, and enhanced market coverage improve responsiveness to market developments and changing environmental conditions, permitting institutions to address circumstances and situations rapidly [12].

In the same article, risks associated with the hub-and-spoke model also were communicated, with these including the following.

1. Congestion at hubs, in cases where patient traffic is routed from one or more spokes without ensuring that main campus hubs can accommodate the noted volume;
2. Overextension of spokes, in cases where spoke-to-hub transit times are too burdensome due to distance or poor transportation infrastructure;
3. Staff dissatisfaction at spokes, in cases where staff members at spokes desire autonomy and grow disenchanted with directives issued by hubs; and
4. Transportation disruptions, in cases where vehicular, roadway, or other elements making up the transportation infrastructure impede spoke-to-hub access [12].

The benefits supplied by the hub-and-spoke organization design are unavailable through competing models of organization, permitting healthcare institutions to stretch each and every dollar of investment in a manner that retains quality, service, and support, despite achieved efficiencies [12, 14–16, 18]. Of course, the network must be designed and managed proficiently in order to capitalize on the model’s associated benefits, requiring obvious care in assembly and operation. While the hub-and-spoke model indeed carries risks, these generally can be minimized or eliminated altogether with proper planning, attention, and action [12].

## Adoption of the model

Willis-Knighton Health System adopted the hub-and-spoke model on the introduction of its first expansion campus, WK South, which opened in 1983. WK South was constructed to complement its sole campus at the time, Willis-Knighton Medical Center, affording the institution a prominent presence in both south and west Shreveport. In the years leading up to this expansion, executives explored organization designs and selected the hub-and-spoke model based on the many benefits that it provided, especially its reputation for efficient and effective service delivery. The model proved to be successful, prompting its continued use in subsequent expansion initiatives. Today, the system’s hub-and-spoke network consists of one hub, Willis-Knighton Medical Center, and five primary spokes: WK South, WK Bossier Health Center, WK Pierremont Health Center, WK Rehabilitation Institute, and The Oaks of Louisiana. Numerous general and specialty medical clinics, located throughout the region, also serve as spokes linked to the main campus hub. The benefits attributed to the hub-and-spoke organization design, as noted earlier in this article, were realized in full by Willis-Knighton Health System, and through the direction of concerted efforts, its associated risks never materialized [11, 12]. While the system established its hub-and-spoke network as a structural platform for operating its own properties, it coincidently and beneficially discovered that the organization design also offered value structurally for the management of relationships with external entities, namely rural hospitals which reached out to the system for support in order to remain viable [11].

## Extension to rural hospitals

With Willis-Knighton Health System’s emergence in the 1980s as a multi-campus institution, its obvious signs of strength garnered attention from other healthcare establishments in the region, including one which was struggling to survive. DeSoto General Hospital, a small community hospital located in Mansfield, Louisiana, situated approximately 37 miles from Shreveport, had been experiencing difficulties which threatened its ability to remain operational. Possessing a legacy of service dating back to 1952, the healthcare facility was encountering the same hardships that many of its peer institutions in sparsely-populated areas faced, with the gravity of urban and suburban centers serving as the catalyst for a downward spiral which impacted community infrastructure and threatened the hospital’s viability. Still, many residents remained in Mansfield and, considering it to be their home, they had no plans to leave. DeSoto General Hospital’s trustees and medical staff members were committed to staying the course and serving the remaining population well, but support was desperately needed to ensure the continued existence of the facility. Looking to an institution of strength and the possible assistance that it could provide, the rural hospital’s leadership approached Willis-Knighton Health System to discuss its interest in a collaborative arrangement which would yield mutual benefits.

Given its focus on market development, Willis-Knighton Health System’s executives were well aware of the plight faced by the region’s rural hospitals. While the system’s growth ambitions were concentrated on locations in Shreveport and neighboring Bossier City, executives understood the role that DeSoto General Hospital played in the continuum of care in northwest Louisiana and entertained the overtures of its leadership to investigate opportunities. Ensuing discussions revealed that the not-for-profit hospital possessed a dedicated governing board and skilled medical staff, but that managerial expertise was desperately needed in order for the facility to capitalize on opportunities and avoid threats in what had become a very challenging environment. Further, the facility needed infrastructure improvements to modernize its appearance and capabilities, permitting it to better serve the wants and needs of the community. It also was imperative for the establishment to capture the healthcare business of the current population, encouraging residents to look to DeSoto General Hospital for healthcare services, something that had become increasingly difficult to do, given the state of infrastructure and the appeal of external markets which enticed some to engage in outshopping for many things including health and medical care. Notably, as DeSoto General Hospital was a treasured asset with a rich history, the community desired retaining ownership of the facility, but trustees were open to other forms of collaboration which would permit all parties involved to benefit.

On considering the possibilities, Willis-Knighton Health System’s recent examination and embracement of the hub-and-spoke organization design offered an avenue of opportunity. Executives believed that by providing managerial leadership and infrastructure improvements, DeSoto General Hospital could effectively serve as a satellite or spoke linked to the main campus hub of Willis-Knighton Medical Center, with operation being virtually identical to that of an owned satellite. As the system had already invested in developing the infrastructure necessary to operate the hub-and-spoke model, bringing DeSoto General Hospital into the network would be a relatively simple task.

After making envisioned investments and associated upgrades modernizing the facility and its offerings, DeSoto General Hospital would provide an array of services more comprehensive than it currently offered, but still less robust than that provided by a major medical center. This was in keeping with the tenets of the hub-and-spoke model whereby satellites offer more limited service arrays than do hubs. This basic, but well-rounded, revised service array at DeSoto General Hospital would permit the residents of Mansfield and vicinity to receive the bulk of their care locally, and when more complex medical interventions were necessary, they could be transferred to Willis-Knighton Health System’s hub for treatment. The region’s transit corridors, especially when supported by Willis-Knighton Health System’s transportation division which included ground and air services, facilitated quick access from Mansfield to Shreveport, providing acceptable spoke-to-hub transit times.

This particular arrangement would see residents of Mansfield and surrounding rural communities not only gain assurances of the continuation of DeSoto General Hospital, but also acquire access to an enhanced array of healthcare services provided locally, with expeditious access to the region’s most advanced health and medical offerings being available through Willis-Knighton Health System. In turn, Willis-Knighton Health System would get the benefit of increased patient volume, drawing on a marketplace removed from its current locations of operation, facilitating its desires to deliver a greater portion of the region’s care. Willis-Knighton Health System also would gain the fulfillment associated with shoring up healthcare services in a community which very likely would lose its hospital if the system did not take action.

Discussions over several months between Willis-Knighton Health System and DeSoto General Hospital eventually resulted in the development of a partnership between the two institutions consistent with the presented characteristics. Daily oversight of the hospital was turned over to Willis-Knighton Health System, investments which upgraded the facility were made, and DeSoto General Hospital was introduced as a partner spoke within the system, with the community retaining ownership and the facility retaining its original brand identity. The partnership was formalized in 1983 and continues to this day, reflecting a very productive, long-lasting arrangement and usefully demonstrating the ability of the hub-and-spoke organization design to bolster rural healthcare.

## Outcomes and implications

The outcomes of a partnership that has now lasted over three decades have been monumental for the citizens of Mansfield and the small communities surrounding the city. In fact, DeSoto General Hospital is now known as DeSoto Regional Health System to better reflect its extended scope of services, thanks largely to Willis-Knighton Health System’s expertise and investments over the years. DeSoto Regional Health System now consists of a 34-bed acute care hospital, three rural primary care clinics, an industrial medicine clinic, and a therapy services clinic and fitness center. For an institution which was on the brink of closing, this represents quite a turnaround, courtesy of the associated partnership between the two entities.

While many possibilities exist for structuring partnerships, Willis-Knighton Health System’s executives viewed its recent adoption of the hub-and-spoke model to have positively impacted decision making and associated efforts which resulted in an improved outcome that otherwise would not have been realized. Specifically, by working to elevate DeSoto General Hospital to function as a partner spoke of Willis-Knighton Health System, its service array had to be upgraded to provide the depth, breadth, and quality required to function fully as a spoke. This necessitated the significant enhancement of health and medical services provided by the hospital, benefiting the populace tremendously and encouraging many who had opted against using their local facility due to poor infrastructure and limited services to once again extend their patronage. The burgeoning patient volume aided DeSoto General Hospital, but it also benefited Willis-Knighton Health System, courtesy of the increased referrals generated from greater patient traffic at the local level. Had Willis-Knighton Health System opted to simply stabilize DeSoto General Hospital, infusing expertise and other resources centered on delivering the facility’s existing service array and treating it as a purely independent operation—a common approach with such partnerships—the outcomes would not have been as robust as those achieved by the facility’s effective transformation into a partner spoke of Willis-Knighton Health System.

Willis-Knighton Health System’s executives also found that the hub-and-spoke model facilitated integration of DeSoto General Hospital into the system’s network, with the model’s attributes encouraging the partnership. As a highly-scalable model, the hub-and-spoke organization design is perfectly suited for adding spokes as warranted to the network with relative ease. Accommodations for increased referrals to the hub indeed must be addressed and associated investments must be made, but by and large, the impact of an additional spoke is negligible on system-wide operations. This has the effect of minimizing downside risk, too. If a given spoke happens to not yield desired benefits, it can be shed with an equally negligible impact. Additionally, as in the case of DeSoto General Hospital which remained externally owned, if a governing board desires to withdraw from partnering at some point in the future, the spoke can be removed from the network with relatively few difficulties. This flexibility, of course, increases openness to engaging in partnerships, potentially leading to more arrangements being effected than would otherwise be the case, something that might hasten the willingness of large healthcare institutions to come to the aid of their struggling rural counterparts.

**Fig. 1:**
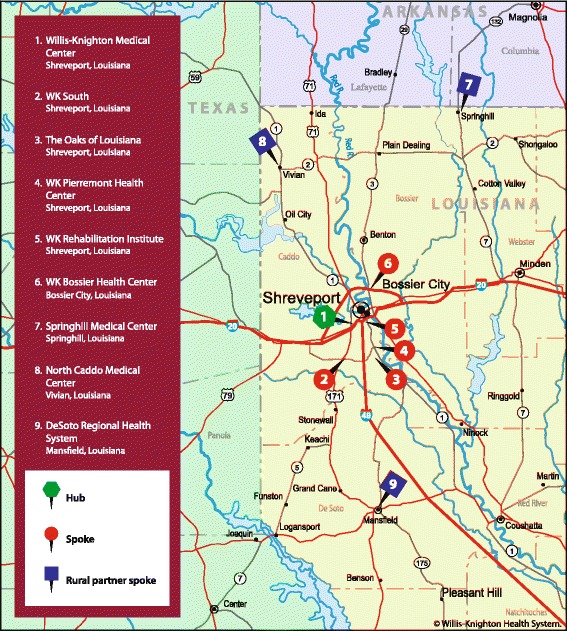
A map presenting Willis-Knighton Health System’s hub-and-spoke network, including its rural hospital partner spokes

Successes realized through the DeSoto General Hospital partnership and the ease of integration and operation afforded by the hub-and-spoke network compelled Willis-Knighton Health System to form two additional partnerships with struggling rural hospitals in northwest Louisiana: North Caddo Medical Center, located in Vivian, Louisiana, situated approximately 33 miles from Shreveport, and Springhill Medical Center, located in Springhill, Louisiana, situated approximately 60 miles from Shreveport. These facilities, too, receive Willis-Knighton Health System’s expertise and investments and operate as partner spokes, which refer intensive cases to the hub for treatment. A map presenting these three rural hospital initiatives in the context of Willis-Knighton Health System’s hub-and-spoke network is provided in Fig. 1. Without Willis-Knighton Health System’s interventions, all three of these rural hospitals likely would have failed, leaving thousands of residents in the remote regions of northwest Louisiana without quick and convenient access to healthcare.

## Conclusions

Given the number of rural hospitals under threat of closure across America, opportunities to restore their viability to ensure that their medically underserved populations continue to have local access to healthcare should not be overlooked. Willis-Knighton Health System found the hub-and-spoke organization design to provide significant utility and value which fostered its receptiveness to support struggling rural hospitals in northwest Louisiana. The resulting partnerships placed the hospitals on firm financial ground and enhanced the depth and breadth of health and medical services provided locally in these small communities, benefiting rural populations and bolstering referrals to Willis-Knighton Health System from each of these partner facilities. As the hub-and-spoke organization design played a key role in making these mutual benefits possible, the model should receive careful consideration by institutions seeking to elevate and enhance the status and stature of healthcare services offered in rural communities.
